# Origin of the right vertebral artery from the right common carotid artery in the setting of an aberrant right subclavian artery: Case and retrospective review to determine frequency

**DOI:** 10.1016/j.radcr.2026.01.002

**Published:** 2026-02-09

**Authors:** David Becker-Weidman, Arthur Parsee, Abraham Ahmed

**Affiliations:** Department of Diagnostic Imaging, Moffitt Cancer Center, 12902 USF Magnolia Drive, Tampa, FL, 33612, USA

**Keywords:** Aberrant right subclavian artery, ARSA, Vertebral artery anomaly, Vascular anomaly, Anatomic variant

## Abstract

Anomalies of the vertebral arteries are rare. The second most common vertebral artery anomaly is the right vertebral artery arising from the right common carotid artery in the setting of an aberrant right subclavian artery (ARSA). We present a case of this rare anomaly and determine the relative frequency with which the right vertebral artery arises from the right subclavian artery vs the right common carotid artery in the setting of an ARSA by retrospectively reviewing over 500 patients with an ARSA and a contrast-enhanced CT. We found that in the setting of an ARSA the right vertebral artery arises from the right subclavian artery in 84% of patients and from the right common carotid artery in 16% of patients.

## Introduction

The right vertebral artery is typically the first branch from the right subclavian artery. Anomalies of the vertebral arteries are relatively rare with the most common being direct origin of the left vertebral artery from the aortic arch which is seen in approximately 5% of people [1,2]. The second most common vertebral artery anomaly is right vertebral artery arising from the right common carotid artery in the setting of an aberrant right subclavian artery (ARSA) which has been reported to be present in 0.18% of people [3].

ARSA is an anomaly where the right subclavian artery arises directly from the aorta distal to the origin of the left subclavian artery instead of arising from the brachiocephalic trunk. Although relatively rare ARSA is the most common anomaly of the major branches of the aortic arch and occurs in approximately 0.5%-1% of people [4,5]. The majority of patients with ARSA are asymptomatic although it can be symptomatic, classically associated with dysphagia [6] (so-called dysphagia lusoria) due to the retroesophageal course of the right subclavian artery as it passes from the left to the right.

**Fig. 1:**
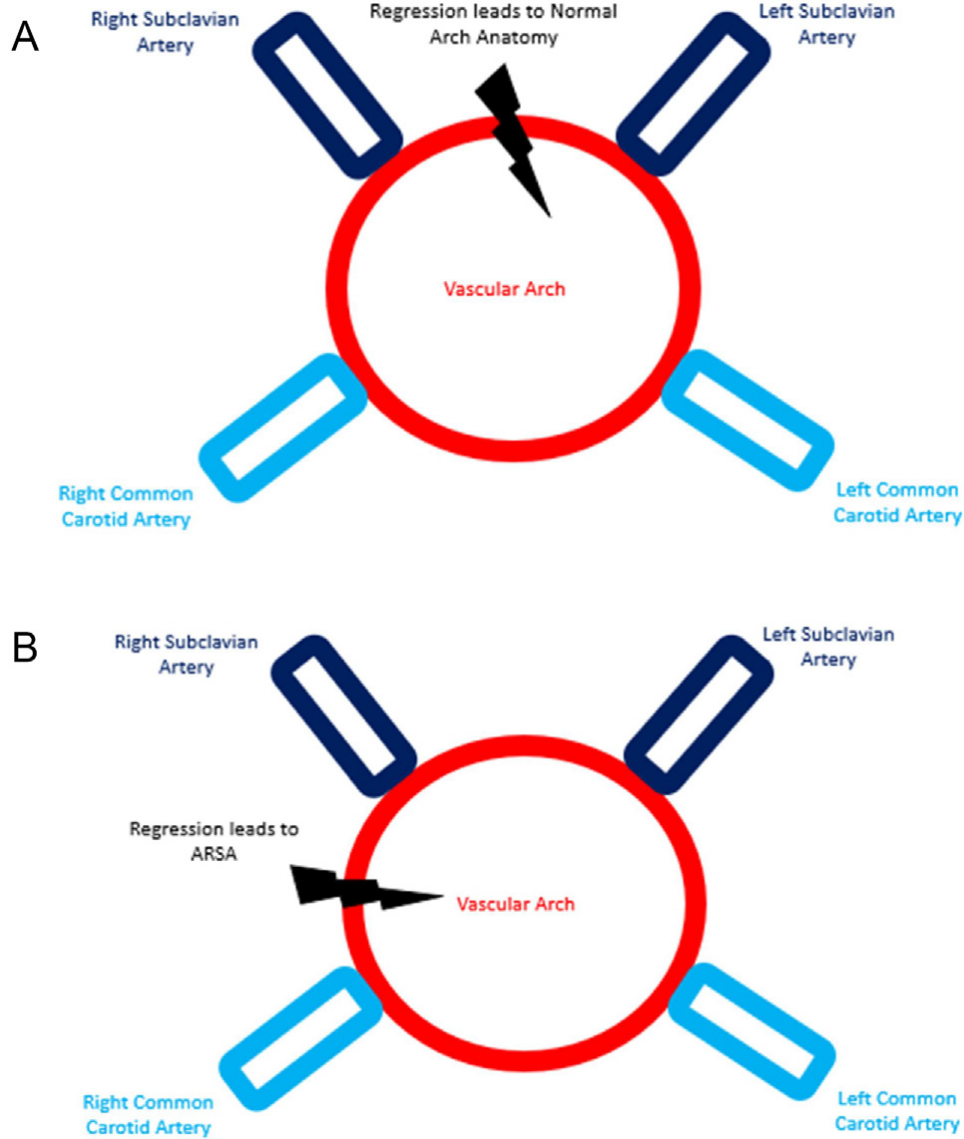
Aortic arch embryology: (A) Regression of the vascular ring separating the right common carotid and right subclavian arteries from the left common carotid and left subclavian arteries leads to the normal aortic arch anatomy and (B) Regression of the vascular ring separating the right common carotid artery from the right subclavian artery leads to an aberrant right subclavian artery.

**Fig. 2:**
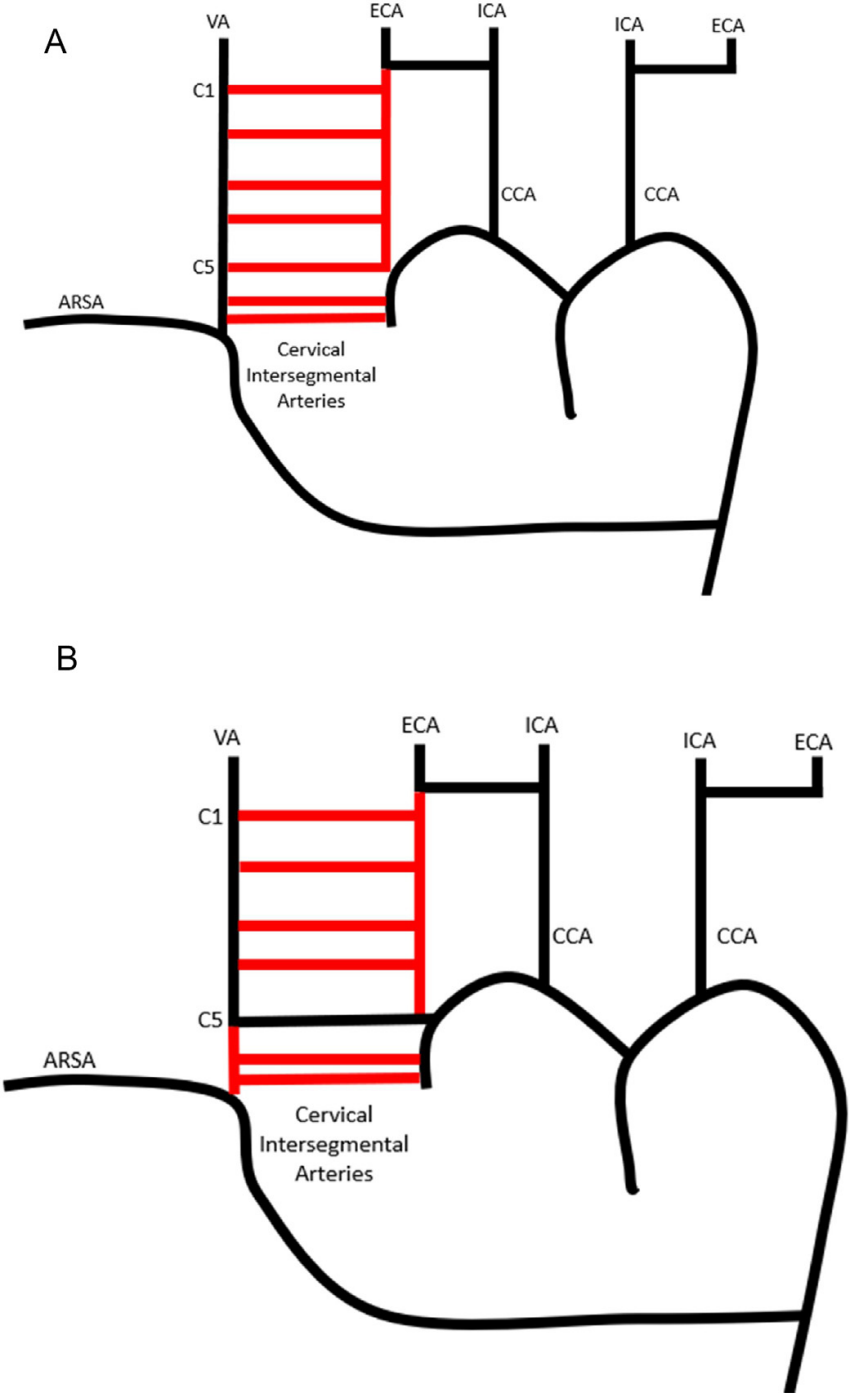
Vertebral artery embryology: (A) Longitudinal anastomosis between the 7 cervical intersegmental arteries with regression (red lines) of the first 6 cervical intersegmental arteries will lead to a right vertebral artery arising from the subclavian artery and (B) Longitudinal anastomosis until the C5 cervical intersegmental artery with persistence of the C5 intersegmental artery will lead to the right vertebral artery will arise from the right common carotid artery as the 1st 4, 6th, and 7th cervical intersegmental arteries will regress (red lines).

The embryologic development of the aortic arch and its major branches involves a series of complex steps. This includes development of vascular ring around the trachea/esophagus which will become the aortic arch with the common carotid arteries and subclavian arteries each arising independently from their respective side of the vascular ring. During normal development the vascular ring dividing the right and left arteries regresses however if the portion of the vascular ring between the right common carotid artery and right subclavian artery regresses it leads to an ARSA [7] (Fig. 1). The vertebral arteries are formed by longitudinal anastomosis between the 7 cervical intersegmental arteries. During normal development the first 6 cervical intersegmental arteries regress and the 7th remains as the proximal subclavian artery and the vertebral artery will arise from the subclavian artery. In the setting of an ARSA the 7th cervical segmental artery is presumably absent or regresses along with the others. If the longitudinal anastomosis ends at the C5 cervical intersegmental artery the C5 intersegmental artery will persist and the vertebral artery will arise from the common carotid artery [7] (Fig. 2).

## Case presentation

**Fig. 3:**
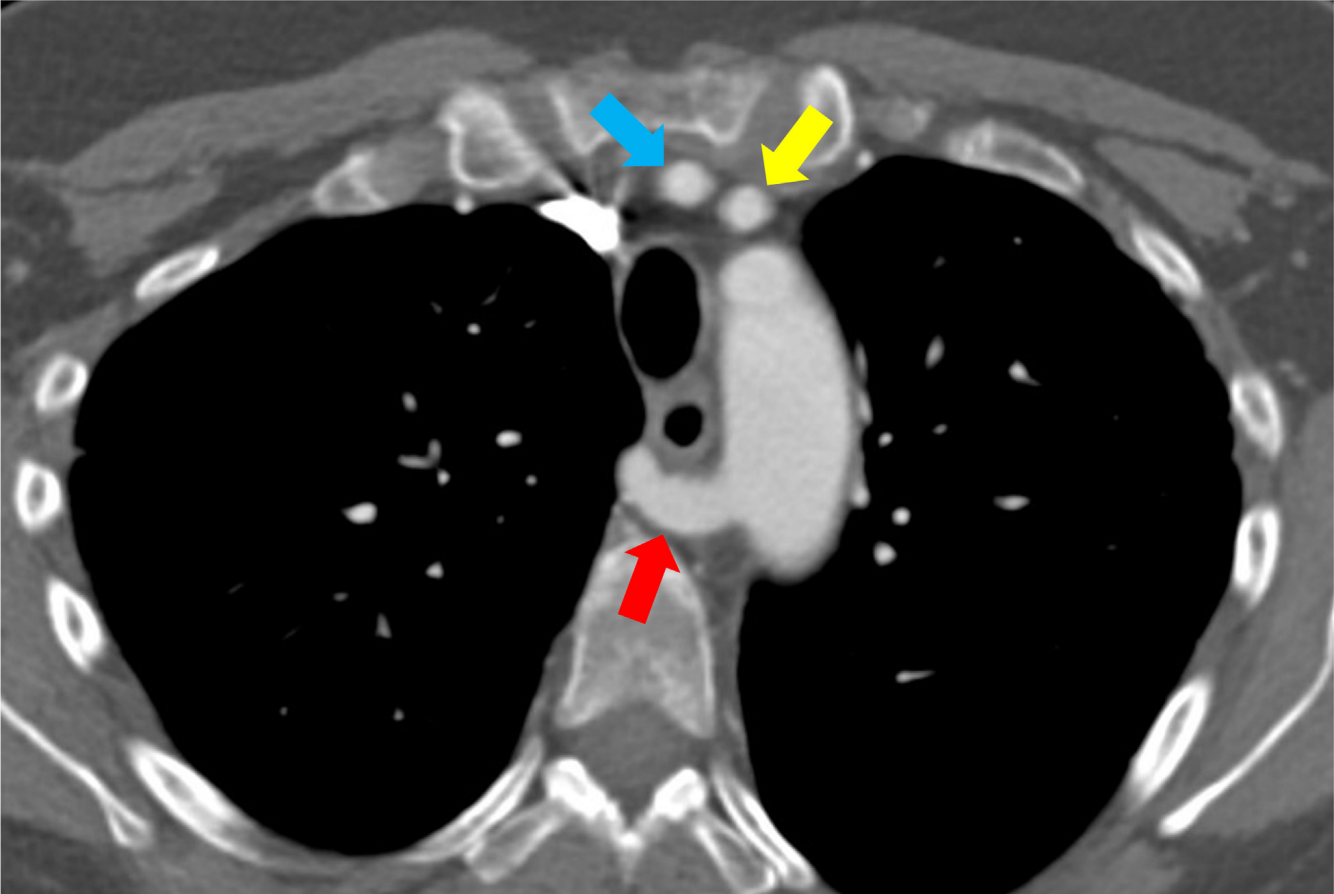
ARSA: Axial CT with contrast at the level of the aortic arch shows the right subclavian artery (red arrow) arising from the distal aortic arch and crossing the midline behind the esophagus. Also seen is the right common carotid artery (blue arrow) and left common carotid artery (yellow arrow) which are the 1st and 2nd branches from the aortic arch in the setting of an ARSA.

**Fig. 4:**
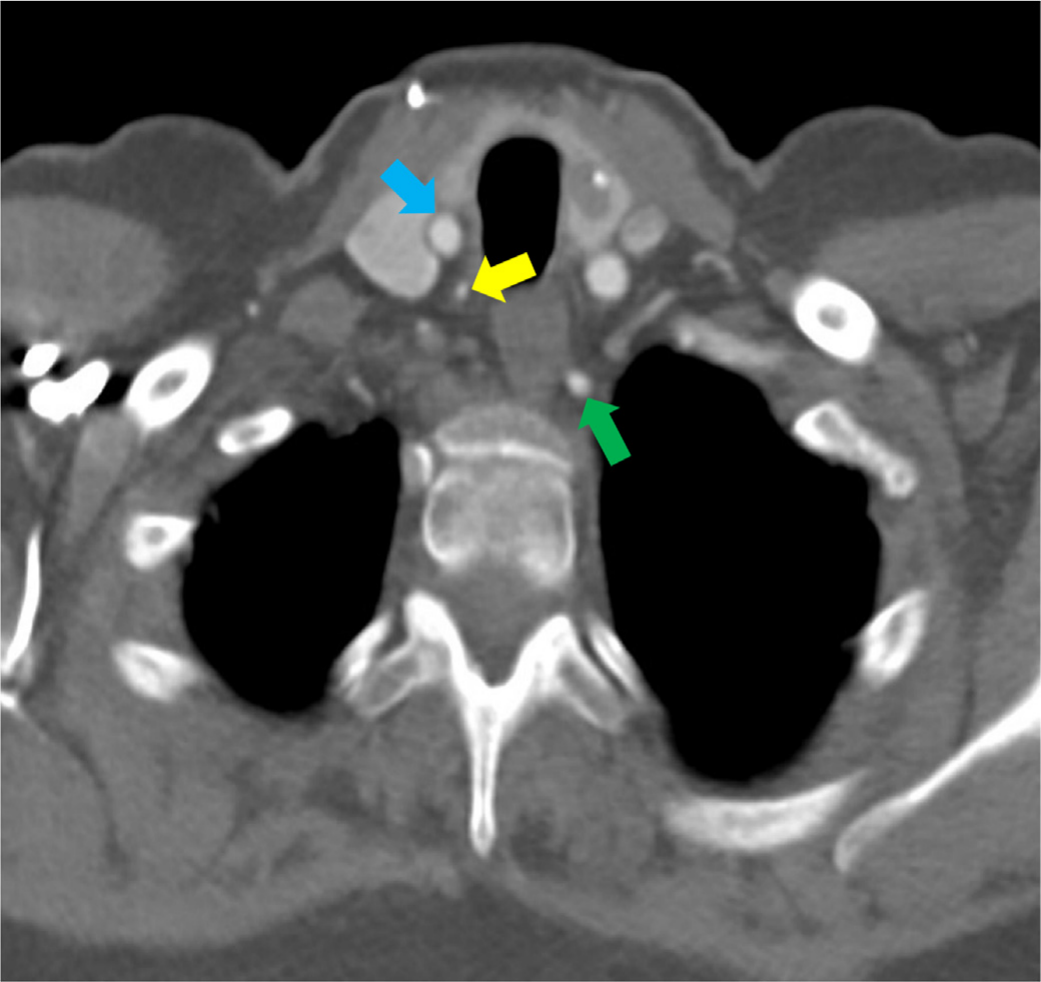
Course of the right vertebral artery at the thoracic inlet: Axial CT near the thoracic inlet shows the right vertebral artery (yellow arrow) next to the right common carotid artery (blue arrow). Also visible is the left vertebral artery (green arrow).

**Fig. 5:**
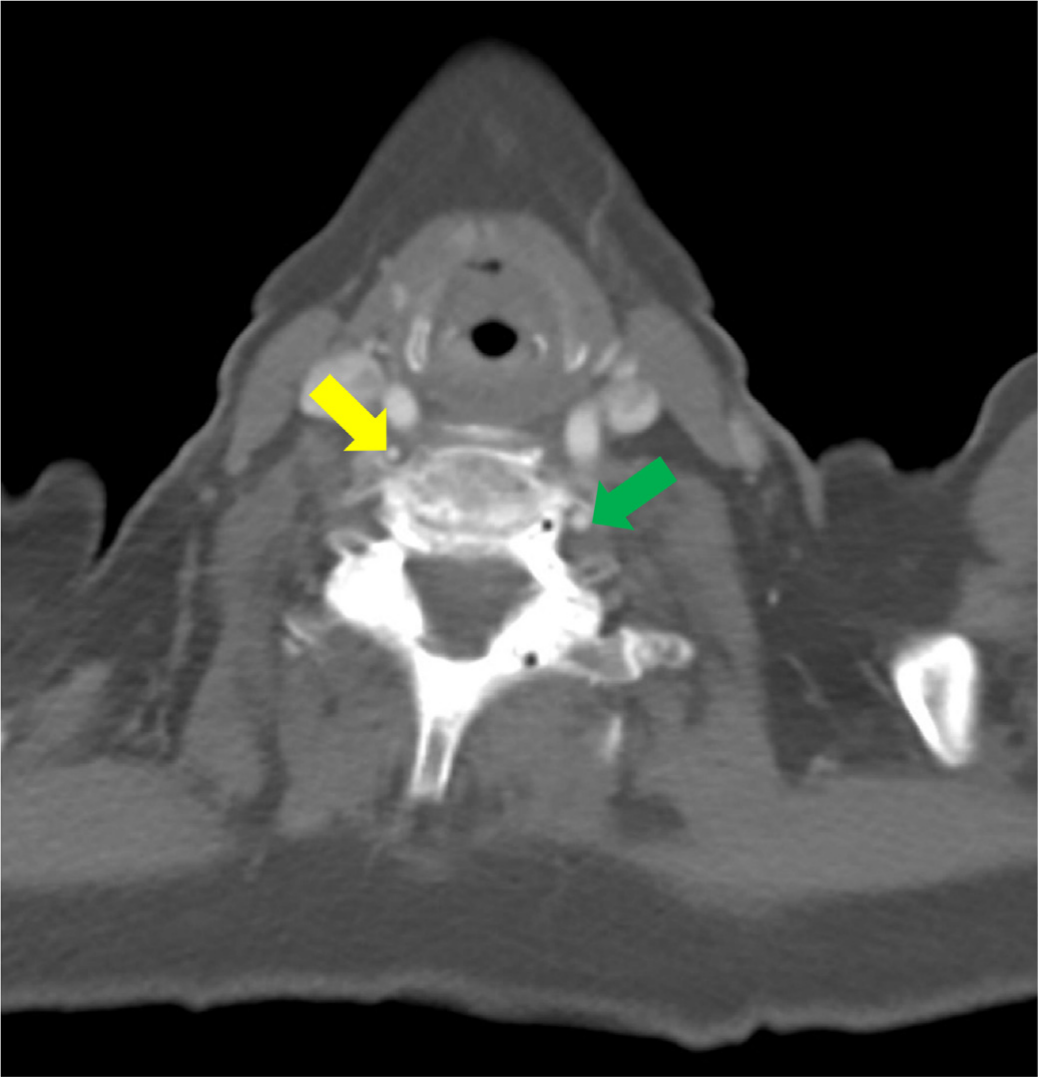
Course of the right vertebral artery in the lower neck. Axial CT at the C6 level shows that the right vertebral artery (yellow arrow) is still anterior to and outside the transverse foramen whereas the left vertebral artery (green arrow) is within the transverse foramen.

**Fig. 6:**
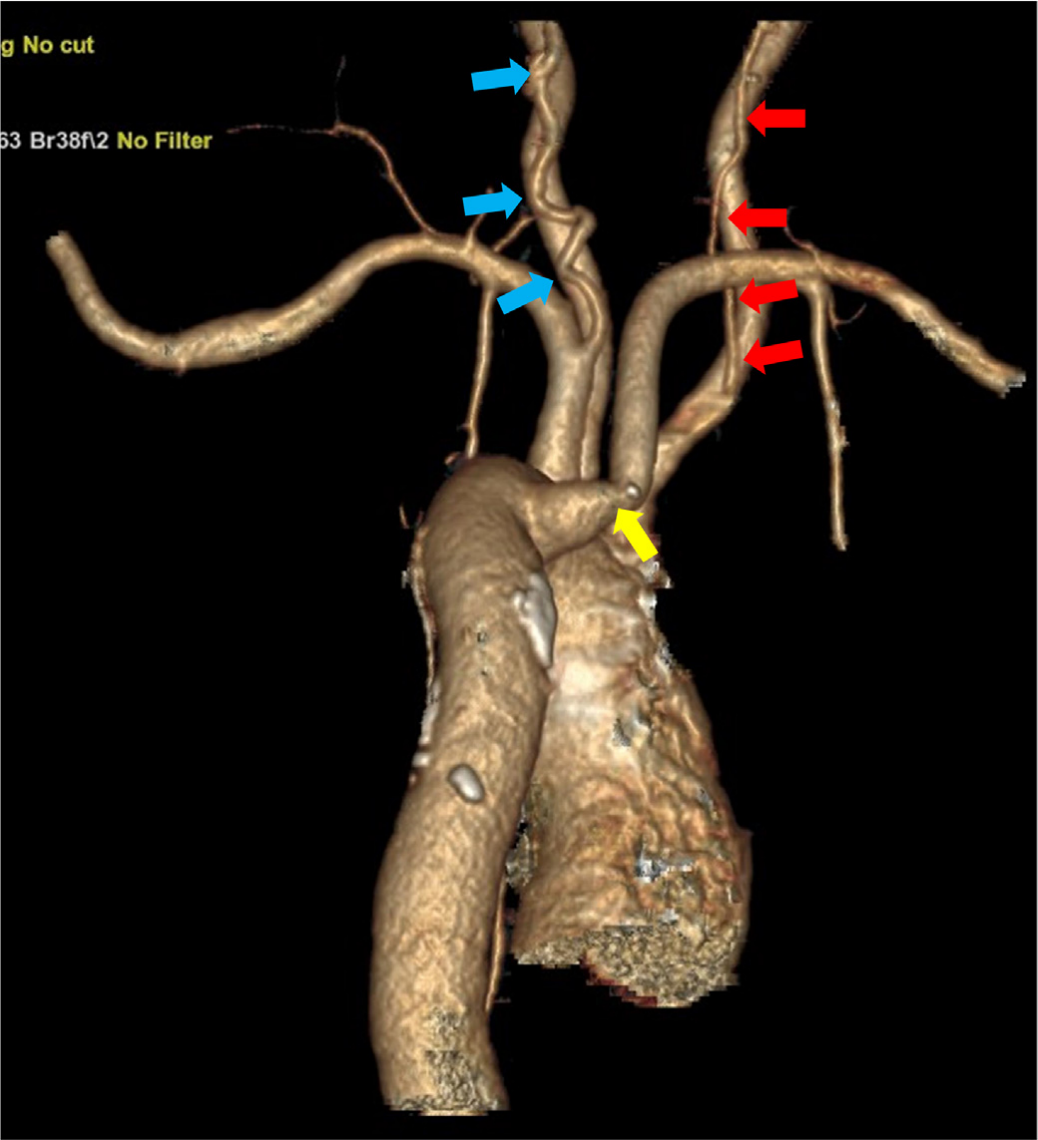
Volume-rendered 3-D images: posterior view of the aortic arch shows the right vertebral artery (red arrows) arising from the right common carotid artery and the left vertebral artery (blue arrows) arising from the left subclavian artery. The aberrant right subclavian artery (yellow arrow) is seen as the last branch from the aortic arch.

65-year-old woman with a subcentimeter neuroendocrine tumor in the pancreatic head undergoing yearly routine surveillance imaging. CT with contrast of the chest, abdomen, and pelvis shows an incidentally-noted ARSA with a retroesophageal course (Fig. 3). Images near the thoracic inlet show the right vertebral artery adjacent to the right common carotid artery because it arises from the right common carotid artery (Fig. 4). Images in the lower neck at the C6 level show the right vertebral artery anterior to the transverse foramen adjacent to the longus colli muscle and the left vertebral artery within the transverse foramen (Fig. 5). The relationship of the right common carotid artery and right vertebral artery may be better appreciated on 3-D volume rendered images (Fig. 6).

## Discussion

After confirming IRB exemption our institutional database of radiology reports was queried using the search terms “aberrant right subclavian artery”, “aberrant origin of the right subclavian artery”, and “ARSA” and filtered to show only CTs with contrast which returned 513 patients. The CTs were reviewed by a body radiologist with 8 years of experience to determine the origin of the right vertebral artery. Of the 513 patients with ARSA and a contrast-enhanced CT the right vertebral artery arose from the right subclavian artery in 433 patients (84%) and the right common carotid artery in 80 patients (16%).

In the setting of typical anatomy the vertebral arteries enter the transverse foramen at the C6 level in the overwhelming majority of cases [8]. We found that the course of the vertebral artery and relationship to the transverse foramen was much more variable in the setting of an origin from the right common carotid artery in the setting of an ARSA. Similar findings were reported by others with entrance levels of C5 [9], C4, C3, and even C2 [10]. This longer extraspinal course of the right vertebral artery is significant as it increases its vulnerability to trauma and can also pose a major danger for cervical spine surgeons if not identified preoperatively.

No anatomic classification system includes the variable origin of the right vertebral artery in the setting of an aberrant right subclavian artery. Celikyay et al. [1] and Ergun et al. [2] both made classifications systems for the variable branching pattern of the aortic arch. Celikyay et al. [1] described 11 branching patterns of the aortic arch and showed the right vertebral artery arising from the right subclavian artery in all 3 aortic arch branching patterns which include an aberrant origin of the right subclavian artery. Ergun et al. [2] described 7 branching patterns of the aortic arch and although they did identify 1 case of the right vertebral artery arising from the right common carotid artery in the setting of an aberrant right subclavian artery it did not figure into their classification scheme.

Similar to the 2 cases reported by Park et al. [9] in our case the right vertebral artery traveled along the anterior surface of the longus colli muscle as it ascended in the neck. This abnormal course has significant clinical implications for anterior cervical spinal surgery and thyroidectomy. The right vertebral artery could be stretched or lacerated during anterior spinal surgery while lateralizing the longus colli muscles to obtain access. The right vertebral artery could be inadvertently ligated during a thyroidectomy noting that due to the abnormal course of the right vertebral artery it will be located in the close vicinity of the inferior thyroid artery which is ligated as part of a thyroidectomy [10]. In addition, right carotid artery procedures are especially risky with this anatomic variant noting the right anterior circulation and right posterior circulation share a common origin which if compromised can lead to catastrophic simultaneous right hemispheric and right brainstem ischemia. Additionally, this anomaly is relevant in the setting of Kommerell diverticulum repair and tracheostomy [11].

## Limitations

There are several limitations to this study which includes the inherent limitations of any retrospective and potential selection biases inherent in the database query. This includes the fact that we are relying on the radiology report to explicitly mention an aberrant right subclavian artery using specific search terms to identify patients for the study. It is inevitable that some patients with an ARSA were excluded from the study due to the radiology report. Finally, only 1 radiologist reviewed the images so inter-observer reliability metrics (eg, Kappa scores) are not available to ensure an accurate and reproducible result.

## Conclusion

In the setting of an ARSA the right vertebral artery arises from the right common carotid artery in 16% of patients. Identifying the origin of the right vertebral artery in the setting of ARSA has significant clinical implications.

## Patient consent

Written informed consent was obtained from the patient to publish their case.
